# A Novel Squirrel Respirovirus with Putative Zoonotic Potential

**DOI:** 10.3390/v10070373

**Published:** 2018-07-18

**Authors:** Leonie F. Forth, Andrea Konrath, Kristin Klose, Kore Schlottau, Kathrin Hoffmann, Rainer G. Ulrich, Dirk Höper, Anne Pohlmann, Martin Beer

**Affiliations:** 1 Institute of Diagnostic Virology, Friedrich-Loeffler-Institut, Südufer 10, 17493 Greifswald-Insel Riems, Germany; leonie.forth@fli.de (L.F.F.); kore.schlottau@fli.de (K.S.); dirk.hoeper@fli.de (D.H.); anne.pohlmann@fli.de (A.P.); 2 Saxon State Laboratory of Health and Veterinary Affairs, Bahnhofstraße 58-60, 04158 Leipzig, Germany; andrea.konrath@lua.sms.sachsen.de; 3 Institute of Pathology, Faculty of Veterinary Medicine, University of Leipzig, An den Tierkliniken 33, 04103 Leipzig, Germany; kristin.klose@vetmed.uni-leipzig.de; 4 Saxon State Laboratory of Health and Veterinary Affairs, Jägerstraße 8/10, 01099 Dresden, Germany; kathrin.hoffmann@lua.sms.sachsen.de; 5 Institute of Novel and Emerging Infectious Diseases, Friedrich-Loeffler-Institut, Südufer 10, 17493 Greifswald-Insel Riems, Germany; rainer.ulrich@fli.de

**Keywords:** novel respirovirus, isolate, pneumonia, Sri Lankan Giant squirrel, potential zoonosis, *Paramyxoviridae*

## Abstract

In a globalized world, the threat of emerging pathogens plays an increasing role, especially if their zoonotic potential is unknown. In this study, a novel respirovirus, family *Paramyxoviridae*, was isolated from a Sri Lankan Giant squirrel (*Ratufa macroura*), which originated in Sri Lanka and deceased with severe pneumonia in a German zoo. The full-genome characterization of this novel virus, tentatively named Giant squirrel respirovirus (GSqRV), revealed similarities to murine (71%), as well as human respiroviruses (68%) with unique features, for example, a different genome length and a putative additional accessory protein. Congruently, phylogenetic analyses showed a solitary position of GSqRV between known murine and human respiroviruses, implicating a putative zoonotic potential. A tailored real-time reverse transcription-polymerase chain reaction (RT-qPCR) for specific detection of GSqRV confirmed a very high viral load in the lung, and, to a lesser extent, in the brain of the deceased animal. A pilot study on indigenous and exotic squirrels did not reveal additional cases in Germany. Therefore, further research is essential to assess the geographic distribution, host range, and zoonotic potential of this novel viral pathogen.

## 1. Introduction

In recent decades, several novel paramyxoviruses have been discovered, which diversified the characteristics of the family *Paramyxoviridae* in terms of genomic structure, genome length, and encoded proteins [1]. Many of them have been isolated from or detected in bats or rodents [2,3,4,5,6]. Nevertheless, the number of known paramyxoviruses in rodents still represents only a small piece of the real paramyxo “virosphere” [7].

The family *Paramyxoviridae*, order *Mononegavirales*, is a large and rapidly growing group of viruses [2]. They are known to cause a variety of respiratory, exanthematous, and neurological diseases in a wide host range. Paramyxoviruses are pleomorphic enveloped viruses containing a linear, non-segmented, negative-stranded RNA genome. To date, the family *Paramyxoviridae* contains seven genera, among them the genus *Respirovirus*, which consists of five species: *Human Respirovirus 1* (HRV1; former *Human Parainfluenza Virus 1*), *Human Respirovirus 3* (former *Human Parainfluenza Virus 3*), *Porcine Respirovirus 1* (former *Porcine Parainfluenza Virus 1*), *Bovine Respirovirus 3* (former *Bovine Parainfluenza Virus 3*), and *Murine Respirovirus* (MRV; former *Sendai virus*) [8].

In 1952, an outbreak of pneumonia in human newborns occurred in Sendai, Japan. A novel virus, initially designated Sendai virus (now MRV), was isolated in laboratory mice that were intranasally inoculated with patient-derived suspensions of the necropsied lungs. The infected mice died after a week with pathological findings, similar to those observed in the deceased human newborns [9]. In the years following, the human origin of “Sendai virus” was controversially discussed, raising the question as to whether MRV had potentially been circulating unnoticed in the laboratory mice population before the inoculation of the human-derived material. This discussion had its basis in the independent detection of MRV in naturally infected mouse breeder colonies [10] and feral house mice [11,12], and was further supported by the fact that no further outbreaks of human disease have been reported since.

MRV is able to cause severe respiratory disease with high mortality in immunocompetent adult mice, being easily transmitted between mice through direct contact or airborne transmission [13]. Other laboratory rodents, such as rats, hamsters, and guinea pigs, can also be infected, however, the infection is generally subclinical or mild. In contrast, HRV1 is one of the common causes of upper and lower respiratory tract infections, especially in children under the age of five years old [14]. Immunity to HRV1 is incomplete and infection can occur throughout life, however, among adults, the disease often occurs in the immunocompromised and elderly [15]. There are currently no licensed vaccines or virus-specific antiviral treatments available [16].

In the present study, we were able to isolate a novel respirovirus, tentatively designated “Giant squirrel respirovirus” (GSqRV), exhibiting sequence similarities to both MRV and HRV1. The virus was identified in a Sri Lankan Giant squirrel (*Ratufa macroura*). Sri Lankan Giant squirrels are distributed throughout Sri Lanka, where it also represents the national animal, and southern India. The International Union for Conservation of Nature (IUCN) lists this species as near threatened as a result of habitat loss and hunting [17]. In addition, we present the characterization of the full-genome sequence, phylogeny, and a pilot monitoring study for the presence of GSqRV in squirrels in Germany.

## 2. Materials and Methods

### 2.1. Sample Collection

The deceased squirrel was dissected and sample materials of different organs were provided for diagnostic purposes. A cold-chain was maintained at all times to ensure the good quality of sample materials. For the routine post-mortem examination, the tissue samples were fixed in 10% neutral buffered formalin (Overlack, Mönchengladbach, Germany) for at least 24 h, embedded in paraffin (Engelbrecht, Edermünde, Germany), sectioned at 3–4 µm, and stained with hemalum and eosin (H.-E.) [18]. The histologic slides were available for histomorphologic examination. Additional tissue samples for screening purposes of 52 Variegated Squirrels (*Sciurus variegatoides*), 27 Prevost’s Squirrels (*Callosciurus prevostii*), 7 Eurasian Red Squirrels (*Sciurus vulgaris*), and 2 Sri Lankan Giant squirrels were previously collected during investigations on Variegated squirrel bornavirus 1 [19,20].

### 2.2. Bacteriological Examination

Bacteriological examination of tissue samples was performed by standard protocols [21] on different solid media (blood and Gassner agar, Thermo Fisher Diagnostics, Waltham, MA, USA) under aerobic, microaerophilic, and anaerobic conditions and on enrichment media (Thermo Fisher Diagnostics) that selectively permit growth of *Salmonella* spp.

### 2.3. Virus Isolation

Tissue homogenates (approx. 10% *w*/*v*) of lung and intestine, and an organ pool consisting of liver, spleen, and kidney samples were used for virus isolation. These samples were prepared by first mincing small pieces of tissue into 1 mm cubes in a sterile Petri dish with dissecting scissors, followed by macerating the tissue cubes further in serum-free culture medium (Hanks’ salts, Biochrom, Berlin, Germany) containing antibiotics (100,000 IU/L penicillin, 100 mg/L streptomycin; Carl Roth, Karlsruhe, Germany) using a mechanical tissue grinder. After incubation for 0.5–1 h at room temperature and centrifugation at 1000× *g* for 15 min, 0.3 mL of the supernatant was inoculated into cell monolayers in duplicates. Virus isolation was performed with freshly prepared confluent monolayers of baby hamster kidney (BHK21, Collection of Cell Lines in Veterinary Medicine (CCLV), Insel Riems, Germany), rabbit kidney (RK13, CCLV), and primary porcine thyroid cells. After inoculation, the cells were incubated at 37 °C for 24 h. Subsequently, the medium containing antibiotics was changed completely and the inoculated cells further incubated at 37 °C for up to one week. Monolayers were monitored microscopically for occurrence of a cytopathic effect (CPE), that is, formation of syncytia. Cultures exhibiting no viral CPE after one week of incubation were additionally passaged twice into freshly prepared cell monolayers before being discarded as negative. The presence of virus was assessed by electron microscopy, hemadsorption, and hemagglutination.

### 2.4. Hemadsorption and Hemagglutination

After incubation for one week at 37 °C, the cell culture monolayers were washed and the medium was replaced by a 0.5% suspension of chicken erythrocytes. After incubation for 1 h at 5 °C, the erythrocytes were washed off, and the monolayers were examined with microscope for evidence of hemadsorption. Hemagglutination titers were determined as described by the regulation of the European Council (92/66/EEC) for Newcastle disease virus.

### 2.5. Electron Microscopy

Cell culture supernatants and homogenized tissue material were negatively stained with 2% phosphotungstic acid (PTA, Riedel-de Haen, Seelze-Hannover, Germany) and examined with a Philips EM 208S transmission electron microscope (Eindhoven, The Netherlands).

### 2.6. Extraction of Nucleic Acids

For sequencing purposes, RNA was extracted using Trizol LS Reagent (Thermo Fisher, Waltham, MA, USA) in combination with RNeasy Mini Kit (Qiagen, Hilden, Germany) including DNase digestion (Qiagen) on the spin column.

Formalin-fixed paraffin-embedded (FFPE) materials of the squirrel including samples of lung, brain, heart, liver, kidney, spleen, and intestine were extracted using the RNeasy FFPE Kit (Qiagen) as described by the manufacturer.

For screening purposes, nucleic acids of squirrel samples were extracted using a combination of Trizol Reagent (Thermo Fisher) and automated extraction with the NucleoMagVet kit (Macherey-Nagel, Düren, Germany). Sample materials were lung, brain, swabs, or organ pools.

### 2.7. Full-Genome Sequencing and Data Analysis

Extracted RNA of the cell culture supernatant (fourth passage) was used for reverse transcription, library preparation, and sequencing of the resulting library lib01567 on a MiSeq instrument (Illumina, San Diego, CA, USA) as previously described [22]. For species identification, the raw data set was first analyzed using the metagenomics data analysis pipeline RIEMS [23]. Subsequently, to obtain the full genome sequence of the identified virus, raw data was quality trimmed and de novo assembled using Newbler (v3.0; Roche, Mannheim, Germany). Variants were determined using the variant finder of the Geneious software suite with minimum variant frequency of 10%, applying parameters of maximum *p*-value of 10^−6^ and filter for strand bias. MAFFT v7.308 [24] was used for nucleotide alignment with subsequent phylogenetic analysis via RAxML v8.2.7 [25], executing 1000 bootstrap inferences and a thorough maximum-likelihood search. Similarity analysis was performed with SimPlot [26] (parameters—window: 200 bp, step: 20 bp, gapStrip: on, Kimura (2-parameter), T/t: 2.0).

The annotated full-length genome sequence of the isolate GSqRV/LKA/2009 has been deposited into the European Nucleotide Archive under the study accession number PRJEB26945.

### 2.8. Real-Time Reverse Transcription-Polymerase Chain Reaction (RT-qPCR)

The full-genome sequence of GSqRV served as basis for the development of a specific RT-qPCR, amplifying a 105 nucleotide (nt) long sequence in the L gene. The oligonucleotide sequences for the specific RT-qPCR were as follows: GSqRV-fw 5′-TCACAGGCTCAAGGATACCG-3′, GSqRV-rv 5′-TCCTTGAGGGCCATGTTGTC-3′, Resp-probe 5′-6-FAM-ACYCAGATGAAGTTCTCHAGTGCMA-CACT-BHQ-1-3′. For a pan rodent respirovirus RT-qPCR (MGSqRV RT-qPCR) simultaneously detecting MRV and GSqRV, primers binding to MRV (MRV-fw 5′-CTGCTGACTCCTGTTTCAAC-3′, MRV-rv 5′-TGTTATRAACCGACTTGCACG-3′) were added. Cycling conditions followed the QuantiTect Probe RT-PCR Kit (Qiagen) manufacturer protocol. For validation, RNA covering the amplification site of MRV and HRV1 was transcribed in vitro using T7-Polymerase (Thermo Fisher) as described by the manufacturer, followed by DNase digestion (TURBO DNA-free™ Kit, Thermo Fisher), and spiked into background squirrel RNA. As an internal control, a primer-probe-system detecting β-actin was applied [27].

## 3. Results and Discussion

### 3.1. Pathological Findings and Initial Pathogen Screening

**Figure 1:**
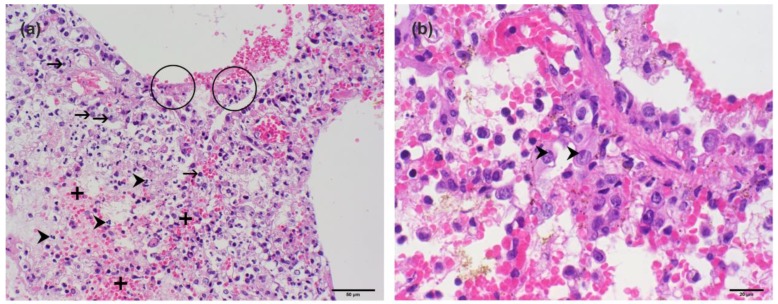
Histopathological findings in the lung of the deceased Sri Lankan Giant squirrel. (**a**) Hemorrhagic-necrotizing pneumonia with group cell necrosis of pneumocytes (circles), acute hemorrhages (plus), and infiltration of neutrophil granulocytes (arrow heads), as well as mononuclear inflammatory cells (arrows); (**b**) eosinophilic intranuclear inclusion bodies in pneumocytes (arrow heads).

In 2009, a male adult Sri Lankan Giant squirrel died during quarantine in a German zoo. The 960-g animal originated from Sri Lanka. Pathologic–anatomic and histologic examinations revealed a moderate to severe acute hemorrhagic-necrotizing pneumonia with intranuclear eosinophilic inclusion bodies, severe diffuse alveolar damage, and multifocal moderate intraalveolar hemorrhages in the lung (Figure 1). Additionally, a moderate mixed-cellular enteritis, dominated by lymphocytes and plasma cells, and multifocal scattered glial nodes in the cerebral cortex, were detected.

Routine diagnostics in virology and bacteriology were performed for identification of the responsible pathogen. Inoculation of homogenized lung material on primary porcine thyroid cells resulted in a CPE including the formation of syncytia on day 3 post inoculation (Figure S1). No CPE was observed after inoculation of homogenized lung material to BHK-21 and RK-13 cell lines during the entire observation period of three blind passages. Inoculation of homogenized materials of intestine and a pool of liver, spleen, and kidney also did not result in a CPE in any of the cell lines.

**Figure 2:**
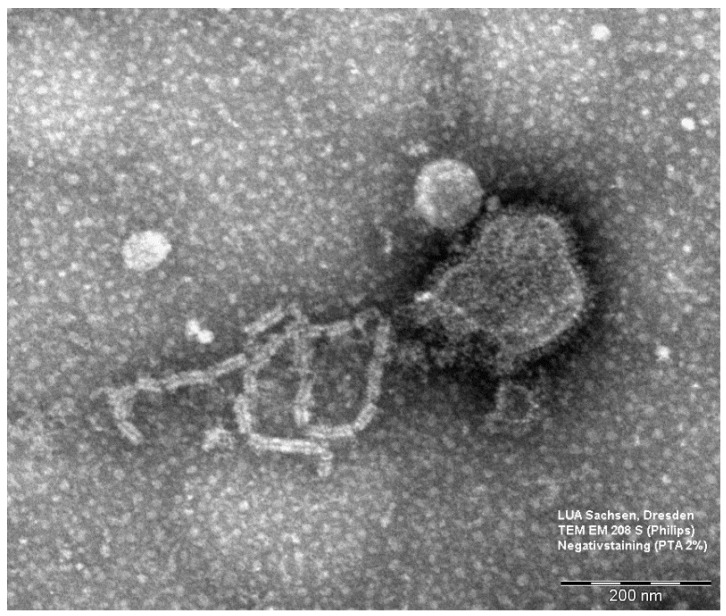
Electron micrograph of Giant squirrel respirovirus (GSqRV) with liberated ribonucleoprotein complex.

Electron microscopy of cell culture supernatant showed typical paramyxovirus-like particles (Figure 2). Hemadsorption and hemagglutination of cell culture supernatant with chicken erythrocytes was positive (HA-titer 1:32). The isolate was named GSqRV/LKA/2009 because of its origin.

Bacteriological examination revealed a high concentration of non-serotypable *Escherichia coli* in the lung, small intestine, and colon, while investigations for *Salmonella* spp. were negative.

To confirm the electron microscopic results and specify the viral species, the obtained raw sequencing data was first analyzed using RIEMS [23]. This initial data analysis of the 2.27 × 10^6^ reads marked approximately each 1% as low quality or unclassified, respectively. Most importantly, with a proportion of 63.8% of the reads being classified as sequences representing the family *Paramyxoviridae*, this analysis confirmed the previous findings but provided no hint at other potentially present pathogens. In addition, 33.8% were eukaryotic sequences, that is, sequences representing the used cell line.

### 3.2. Viral Genome Loads in Different Organs of the Sri Lankan Giant Squirrel

On the basis of the full-genome sequence (generated by next-generation sequencing; see below), a specific RT-qPCR was developed for GSqRV detection using specific primers amplifying a fragment of the L-gene. The viral load in the lung tissue of the squirrel was high, as observed in both the untreated (C_q_ 16), and FFPE material (C_q_ 27). The untreated brain material was also positive (C_q_ 27), whereas in the FFPE material, no viral RNA could be detected. Equally, in the FFPE material of heart, liver, kidney, spleen, and intestine, no viral RNA could be detected. The strong presence of viral RNA in the lung points to GSqRV being accountable for the hemorrhagic-necrotizing pneumonia. The detection of GSqRV RNA in the brain material, although at a much lesser extent, indicates a severe systemic infection with GSqRV, potentially causing the observed multifocal scattered glial nodes. Acute encephalopathy associated with respiroviruses has also been described for some human cases upon infection with HRV1, although extra-pulmonary symptoms are rare [28,29].

### 3.3. Genome Analysis and Features of GSqRV

#### 3.3.1. Genome Organization

The full-genome sequence of isolated GSqRV/LKA/2009 was obtained by random primed cDNA synthesis followed by shot-gun sequencing, with subsequent de novo assembly of the raw data (mean depth >1000). A unique feature of GSqRV is the genome length of 15,378 nucleotides (nt), complying to the rule of six for members of the *Paramyxoviridae* family, which is crucial for efficient genome replication [30]. In contrast, MRV genomes are 6 nt longer with a length of 15,384 nt, while HRV1 strains have a genome length of 15,600 nt.

The genome comprises the six genes 3′-N-P/C-M-F-HN-L-5′. The localization and length of the coding sequences (CDS) for the nucleoprotein (N), phosphoprotein (P), the accessory proteins (C, C’, Y1, Y2), and the matrix protein (M) are analogous to the genome organization of MRV. In contrast, the CDS for the fusion protein (F), hemagglutinin–neuraminidase (HN), and polymerase (L) differ in their positions compared with MRV and HRV1 because of six missing nucleotides in the intergenic region between M and F (Table 1). The CDS for the F protein is, in comparison with MRV and HRV1, extended by 24 nt and 54 nt, respectively, leading to the expression of a hypothetically larger fusion protein. The HN protein of GSqRV is supposedly one amino acid residue shorter.

#### 3.3.2. Putative Additional Accessory Protein

**Figure 3:**
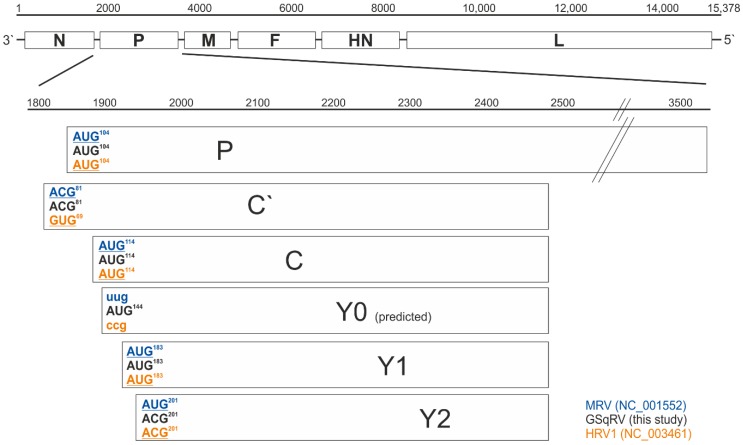
Schematic representation of the coding sequences (CDS) located in the P gene including the new open reading frame (ORF) Y0 in the GSqRV genome. The P mRNA transcript serves as template for the synthesis of the phosphoprotein and the accessory proteins located in the +1 ORF, accessible through a ribosomal scanning mechanism. The superscript numbers indicate the position of the first base of the start codon relative to the 5′ end of the mRNA; blue: murine respirovirus (MRV), strain Nagoya (NC_001552), black: GSqRV (this study), orange: human respirovirus 1 (HRV1), strain Washington/1964 (NC_003461); underlined: protein expression was verified by others [39,41,42], lower case: non-start codons at the corresponding position.

As a unique feature of GSqRV, an additional open reading frame (ORF) is located in the P gene, leading to the hypothetical expression of an additional accessory protein (Y0), not present in MRV and HRV1. The putative fifth translation site is located in the P gene, upstream of the initiation sites for Y1 and Y2 (Figure 3). The expression of the potential accessory protein Y0 could result in a shift in expression levels, as the start codons are ranked in sequence context for initiation [31,32]. The putative additional accessory protein is located in a relatively poor Kozak context, except for the purine, a G in this case, at the third nucleotide position upstream of the ATG codon, which, according to Kozak, is the most highly conserved position for initiation of translation in vertebrates [33]. However, the Kozak context might not be of great importance for expression of Y0. It was shown that MRV accessory proteins Y1 and Y2 are expressed by a ribosomal shunting mechanism [34,35], which might also be used for the expression of Y0 and has to be tested experimentally. In general, it has been shown that the P gene encodes for a nested set of additional accessory proteins (C’, C, Y1, Y2), whose expression is mediated by a ribosomal scanning mechanism and internal initiation at different start codons of the mRNA [30]. The start codons for the accessory proteins are located in the +1 reading frame relative to the phosphoprotein-encoding reading frame of the P gene. In some cases, alternative start codons (ACG, GUG), instead of AUG, are found as initiation site for expression of accessory proteins, as is also the case with GSqRV (Figure 3). The established accessory proteins were shown to be important for efficient production of infectious particles [32,36], as well as for the regulation of viral mRNA synthesis [37,38] and counteracting the host cell interferon responses [39,40].

#### 3.3.3. RNA Editing

Within MRV, the P gene serves also as a template for the proteins V and W, being produced by a leaky scanning mechanism of the polymerase, and thereby insertion of non-templated G nucleotides into the mRNA, resulting in a shift of the reading frame [43]. The resulting putative V and W proteins display amino-terminal similarity with the P protein, while carboxy-terminally, the amino acid sequence differs because of the G insertion-mediated frameshift. The function of these proteins has not been finally elucidated, but it has been shown that the V protein has an effect on viral genome replication, pathogenicity, and inhibition of interferon-β production [44,45,46]. Because the consensus sequence motif necessary for RNA editing (3′-UUUUUUCCC-5′) [45] is also present in the GSqRV genome, it can be assumed that V and W proteins will also be expressed. In contrast, HRV1 does not express V and W proteins because of the missing signal for RNA editing [47].

#### 3.3.4. Single Nucleotide Variants (SNVs)

In the genome sequence of the GSqRV, 13 SNVs were detected (cutoff = 10%), suggesting the existence of at least one variant. Four SNVs are located in the HN-CDS, and an additional four in the L-CDS (see Table S1). Only 2 of 13 polymorphisms are non-synonymous, with one of them affecting the overlapping CDS for P and the accessory proteins, resulting in an exchange in all these proteins. The other is a two nucleotide polymorphism-mediated amino acid exchange in a HN protein region of high structural similarity, which is probably located in its globular head [48].

### 3.4. Sequence Identities to Other Paramyxoviruses

**Figure 4:**
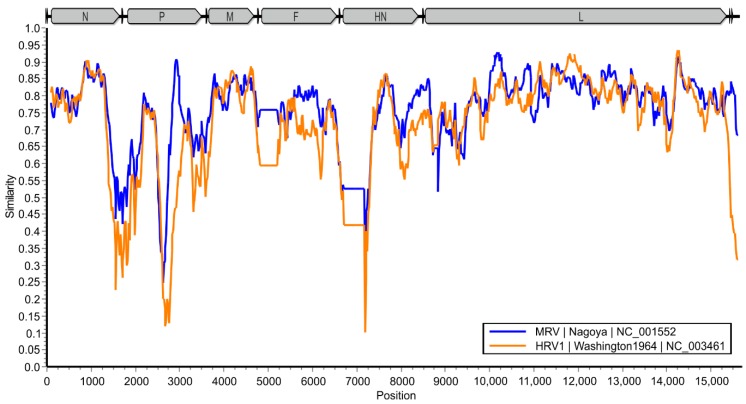
Similarity analysis of the GSqRV genome to MRV and HRV1 using SimPlot. The sequence identity within a sliding window of 200 nt centered on the plotted position is depicted, with a step size of 20 nt.

The overall pairwise nucleotide sequence identity of the GSqRV is only slightly higher to MRV (71%, NC_001552) than to HRV1 (68%, NC_003461), while the sequence identities within a sliding window of 200 nt differ from 11 to 94% (Figure 4).

The amino acid sequence identities vary between 55% and 89% to MRV, strain Nagoya, and between 49% and 87% to HRV1, strain Washington/1964, respectively (Table S2). The M gene and M protein have the overall highest sequence identities to MRV and HRV1 at nucleotide (77% and 76%, respectively), and amino acid level (89% and 87%, respectively). In contrast, the P gene and P protein exhibit the lowest sequence identities at nucleotide (67% and 62%, respectively) and amino acid level (55% and 49%, respectively). These results fall in line with the general observation that within the former subfamily *Paramyxovirinae,* the sequence similarity between corresponding proteins is greater for N, M, and L, with F and HN being slightly less conserved, and C and P being poorly conserved [49].

To the best of our knowledge, no other squirrel-specific respiroviruses have been reported in the literature or the sequence databases to date. There has been serological evidence of MRV in wild grey squirrels in North Wales, however, in this study, no virus was characterized [50]. In another study, the authors reported on the isolation of paramyxoviruses from the kidneys of grey squirrels [51]. For these Pentland’s paramyxoviruses, which are still unclassified according to the International Committee on Taxonomy of Viruses (ICTV) [52], only three short partial polymerase gene sequences are available. The nucleotide sequence identities between the present GSqRV and these partial sequences are less than 62%.

### 3.5. Phylogenetic Analysis

**Figure 5:**
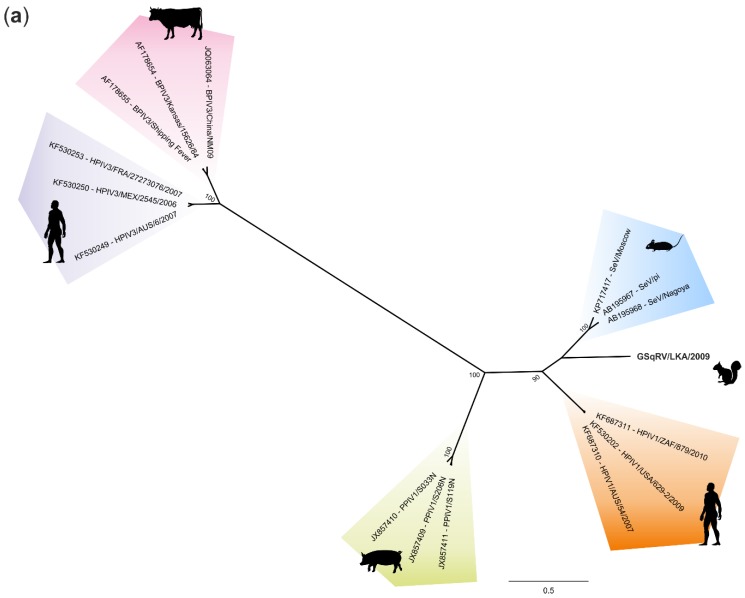

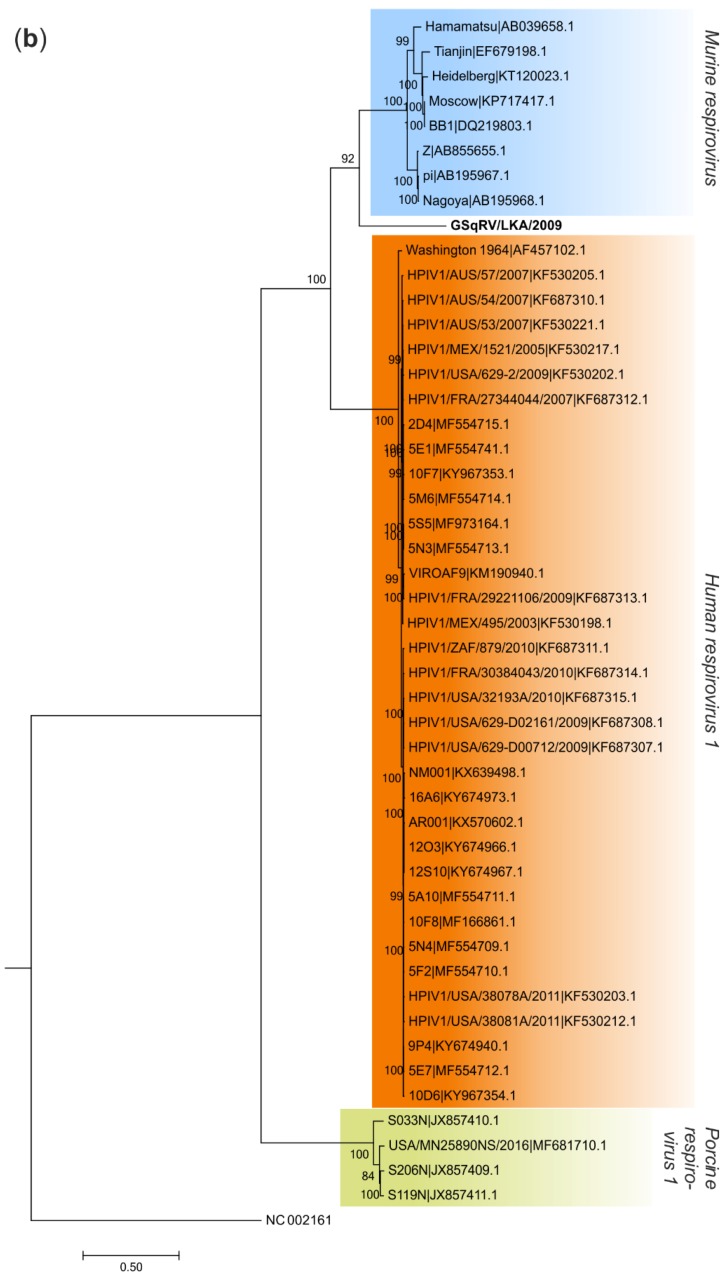
Classification of GSqRV in the genus *Respirovirus* by phylogenetic analysis of selected representatives of each species with the main host depicted in black silhouettes (**a**) and phylogenetic analysis of murine, human, and porcine respirovirus 1 with bovine respirovirus 3 (NC002161) included as outgroup (**b**). Selected were available complete genome sequences within the species excluding clones and revertants. Sequences were aligned with MAFFT and phylogenetically analyzed using RAxML with the model GTR GAMMA and 1000 replicates for bootstrap support. Annotation includes strain/isolate and accession number.

Sequence alignment with subsequent phylogenetic analysis shows that GSqRV can be classified within the genus *Respirovirus*, most closely related to MRV and HRV1 (Figure 5). The outstanding position between MRV and HRV1 in the full-genome-based phylogenetic tree raises the question of affiliation of GSqRV to a certain already defined species (most likely MRV), or to define a new species overall. According to the demarcation criteria for species of the genus *Respirovirus* in the 9th ICTV report [49], each virus species should represent a significant pathogen in its respective host, which is given for MRV and HRV1, and might also be applicable to GSqRV. MRV and HRV1 “have considerable sequence relatedness and antigenic similarity, but also can be clearly distinguished on either basis; also, HRV1 lacks editing and a V protein” [49]. Because stringent cut-off values based on nucleotide or amino acid sequence identities are not defined, as it is the case for, for example, demarcation of lyssavirus species [49], the question of affiliation to, or definition of, a new species is tentatively left open.

### 3.6. Pilot Screening for MRV and GSqRV in Squirrels

For identification of potential further cases in Germany, squirrels living wild or in captivity were screened via RT-qPCR. For simultaneous detection of MRV and GSqRV, a pan rodent respirovirus RT-qPCR assay was developed and applied. Sample materials consisted of lung, brain, oral swabs, or organ pools. In 88 squirrels tested, comprising 52 Variegated Squirrels*,* 27 Prevost’s Squirrels, and two Sri Lankan Giant squirrels from holdings in Germany, as well as seven wildlife Eurasian Red Squirrels, neither GSqRV nor MRV were detected. Therefore, GSqRV infections in squirrels might be very rare in Germany, however, the number of animals in this study was quite limited. Additionally, the natural host of GSqRV is not known. The deceased squirrel could embody the reservoir host or an accidental host. Because of acute stress caused by transportation and adaptation to a new environment, it may have succumbed to the disease, while being unaffected in its natural habitat. The examination of more samples of different rodents is necessary, starting in Sri Lanka and south India, to determine the reservoir, host range, and geographic distribution of GSqRV. Future studies should also include serological analyses, as the viremic phase is probably short and direct detection of viral RNA is thus restricted.

### 3.7. Evaluation of GSqRV as a Potential Zoonotic Agent

The genome similarity of GSqRV and MRV/HRV1 raises concerns of the potential for zoonotic and interspecies transmission of GSqRV. Both MRV and HRV1 are known to be pathogenic for their respective hosts. However, while HRV1 replicates poorly and is nonpathogenic in mice, MRV has been shown to replicate as efficiently as HRV1 in the upper and lower respiratory tract of African green monkeys, and may therefore lack host range restriction [53]. Similarly, the first MRV cases in Sendai suggested a zoonotic potential, although the origin of the virus could not be finally resolved. Taking these findings into account, the replication of GSqRV in primates/humans seems possible, whereas the pathogenicity cannot be predicted. At the serological level, cross-reactivity of antibodies against MRV and HRV1 exists, especially mediated by conserved parts of the N protein [54]. Because of the genetic and most likely antigenic similarity of GSqRV to MRV and HRV1, a cross-reactivity of antibodies against GSqRV seems likely. The cross-reactivity of antibodies against MRV and HRV1 initially led to the consideration of MRV as a potential live-attenuated vaccine against HRV1 [55,56], although this was considered controversial because of the observation that MRV replicates as efficiently as HRV1 in nonhuman primates [53]. Taking this into account, studies of GSqRV as an “intermediate” between MRV and HRV1 may add useful knowledge in regard to vaccine development. Extensive studies based on novel diagnostic methods are needed to assess the zoonotic potential of GSqRV, and future studies would also benefit from experimental studies in potential host species.

## 4. Conclusions

Here, we were able to detect and characterize a novel virus isolated from a deceased Sri Lankan Giant squirrel with severe pneumonia that was imported to Germany. The unique position of the virus in phylogenetic analysis as an “intermediate” between MRV and HRV1 is remarkable and demonstrates the need for further research regarding its zoonotic potential. An initial pilot screening of indigenous and exotic squirrels living in Germany by RT-qPCR did not identify further cases, nevertheless, more squirrels in different areas of the world should be examined to provide more information on host specificity, transmission, and geographic distribution of this novel virus. These investigations will expand our knowledge about wildlife associated viruses that might represent a threat to human and animal health.

## Figures and Tables

**Table 1:** Comparison of positions and lengths of the coding sequences of Giant squirrel respirovirus (GSqRV) with murine respirovirus, strain Nagoya (MRV Nagoya, NC_001552) and human respirovirus 1, strain Washington/1964 (HRV1 Washington, NC_003461). Differences from GSqRV are highlighted in bold.

	Function	MRV (Nagoya)			GSqRV (Sri Lanka)			HRV1 (Washington)		
		Start	Stop	Length	Start	Stop	Length	Start	Stop	Length
N	RNA-binding protein	120	1694	1575	120	1694	1575	120	1694	1575
C’	Accessory protein	1821	2468	648	1821	2468	648	**1809**	2468	**660**
P	Phosphoprotein	1844	3550	1707	1844	3550	1707	1844	3550	1707
C	Accessory protein	1854	2468	615	1854	2468	615	1854	2468	615
Y0	Putative accessory protein	-	-	-	1884	2468	585	-	-	-
Y1	Accessory protein	1923	2468	546	1923	2468	546	1923	2468	546
Y2	Accessory protein	1941	2468	528	1941	2468	528	1941	2468	528
M	Matrix protein	3669	4715	1047	3669	4715	1047	3669	4715	1047
F	Fusion protein	**4866**	**6563**	**1698**	4860	6581	1722	**5088**	**6755**	**1668**
HN	Attachment protein	**6693**	**8420**	**1728**	6687	8411	1725	**6903**	**8630**	**1728**
L	Large polymerase protein	**8556**	**15,242**	6687	8550	15,236	6687	**8772**	**15,443**	**6672**

## Supplementary Materials

The following Supplementary Materials are available online at http://www.mdpi.com/1999-4915/10/7/373/s1, Figure S1: Cytopathic effect of GSqRV on primary porcine thyroid cells, Table S1: Detected sequence variants in the GSqRV genome, Table S2: Sequence alignment identities of individual GSqRV CDS to MRV (NC_001552) and HRV1 CDS (NC_003461).
