# Supplementary material for: A Novel Squirrel Respirovirus with Putative Zoonotic Potential
Source: Viruses. 2018 Jul 18;10(7):373. doi: 10.3390/v10070373 (PMC6070802; doi:10.3390/v10070373)
Supplement: Supplementary file 1 [file viruses-10-00373-s001.pdf]

Supplementary Materials

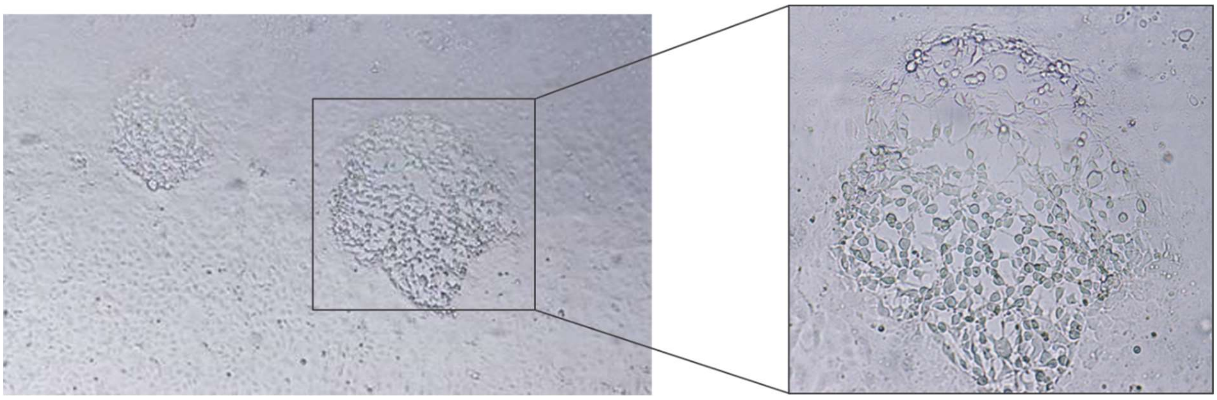

Figure S1. Cytopathic effect of Giant squirrel respirovirus (GSqRV) on primary porcine thyroid cells.

Table S1. Detected sequence variants in the Giant squirrel respirovirus (GSqRV) genome. The cut-off for determining reliable variants was set at a variant frequency of 10%.

Position	Affected CDS	Nucleotide Exchange	Amino Acid Exchange	Variant Frequency [%]	Variant P-Value
989	N	A -> G	no	33.4	0.0
2145	P	T -> C	V -> A	35.0	0.0
2145	C′, C, Y0, Y1, Y2	T -> C	Y -> H	35.0	0.0
2572	P	A -> G	no	35.4	0.0
7338-7339	H	AA -> GC	N -> A	34.0	0.0
7368	H	C -> T	no	35.2	0.0
8078	H	C -> T	no	14.4	2.2E-292
8216	H	G -> A	no	48.1	0.0
9491	L	G -> A	no	33.0	0.0
10,026	L	C -> T	no	30.6	0.0
10,262	L	G -> A	no	30.8	0.0
12,578	L	A -> C	no	33.1	0.0

Table S2. Sequence alignment identities of individual GSqRV coding sequences (CDS) to murine respirovirus (MRV, strain Nagoya, NC_001552) and human respirovirus 1 (HRV1, strain Washington/1964, NC_003461) CDS .

	**Nucleotide sequence identity [%] to**		**Amino acid sequence identity [%] to**	
**CDS**	**MRV**	**HRV1**	**MRV**	**HRV1**
N	71.6	71.2	75.3	69.0
C'	73.0	69.0	72.6	66.2
P	66.6	62.2	55.4	48.5
C	73.0	69.8	72.5	67.6
Y1	74.8	70.9	73.5	71.3
Y2	74.7	70.1	74.3	70.3
M	77.0	76.2	88.8	86.5
F	68.6	63.0	75.4	64.9
HN	68.2	65.5	75.3	69.0
L	74.4	73.7	87.5	83.5
